# Genome Sequencing of Escherichia coli and Klebsiella pneumoniae Isolates That Harbor the FOX-5 β-Lactamase Gene

**DOI:** 10.1128/MRA.01116-20

**Published:** 2020-11-05

**Authors:** Tracy H. Hazen, J. Kristie Johnson, Anthony D. Harris, David A. Rasko

**Affiliations:** a Institute for Genome Sciences, University of Maryland School of Medicine, Baltimore, Maryland, USA; b Department of Microbiology and Immunology, University of Maryland School of Medicine, Baltimore, Maryland, USA; c Department of Pathology, University of Maryland School of Medicine, Baltimore, Maryland, USA; d Department of Epidemiology and Public Health, University of Maryland School of Medicine, Baltimore, Maryland, USA; Indiana University, Bloomington

## Abstract

We generated draft genome assemblies of three Escherichia coli and seven Klebsiella pneumoniae isolates that harbor the FOX-5 β-lactamase-encoding gene.

## ANNOUNCEMENT

The FOX β-lactamases confer resistance to cephalosporins and cephamycins, and the *bla*_FOX_ genes encoding these β-lactamases have been identified on plasmids (1). In a previous study, we described a *bla*_FOX-5_-containing IncA/C plasmid that was identified among Escherichia coli and multiple species of *Klebsiella* from patients in an intensive care unit (ICU) (2). Samples were collected under University of Maryland School of Medicine protocol HP-00066759. We have previously described the genome assemblies of 11 *bla*_FOX_-containing E. coli and *Klebsiella* species isolates (2 – 4), and in the current study, we describe the genomes of 10 additional isolates to augment the data from our previous study (2).

Genomic DNA for whole-genome sequencing was extracted from each of the E. coli and K. pneumoniae isolates as previously described (2). The bacteria were grown overnight at 37°C with shaking in lysogeny broth, and genomic DNA was extracted using the Sigma GenElute bacterial kit following the manufacturer’s protocol. The sequencing libraries were generated with the KAPA HyperPrep library kit (catalog number KK8504) and sequenced on the Illumina HiSeq 2500 platform using a 101-bp paired-end kit.

Raw sequencing reads were filtered to remove the contaminating phiX sequencing control using BBDuk of the BBTools software suite (https://sourceforge.net/projects/bbmap/). The raw reads were also filtered to remove contaminating Illumina sequencing adaptors and quality trimmed using Trimmomatic v.0.36 (5). The resulting filtered reads were assembled using SPAdes v.3.14.1 (6), using the isolate assembly option for high-coverage bacterial isolate data. The assemblies were filtered to contain only contigs longer than 500 bp and a k-mer coverage of ≥5×.

Relevant statistics, including GenBank accession numbers for assemblies and SRA links to the sequencing read data, are included in Table 1. The genome sequences have a mean sequence coverage of 144.9× (standard deviation [SD], 29.22×; minimum [min], 103.07×; maximum [max], 215.86×). The assemblies have a mean contig count of 87 (SD, 24.76; min, 59; max, 139). The E. coli assemblies have a mean size of 5,254,785 bp (SD, 52,213 bp; min, 5,194,917 bp; max, 5,290,892 bp), a mean GC content of 50.63% (SD, 0.21%; min, 50.46%; max, 50.86%), and a mean *N*_50_ value of 195,238 bp (SD, 31,914 bp; min, 166,652 bp; max, 229,671 bp). The K. pneumoniae assemblies have a mean size of 5,678,549 bp (SD, 141,300 bp; min, 5,525,694 bp; max, 5,929,747 bp), a mean GC content of 57.02% (SD, 0.22%; min, 56.56%; max, 57.18%), and a mean *N*_50_ value of 180,006 bp (SD, 34,495 bp; min, 111,734 bp; max, 218,486 bp). The genomes were annotated with PGAP v.4.12 (7).

**TABLE 1:** Genome metrics^*a*^

Species	Isolate	MLST^*b*^	No. of contigs	Assembly size (bp)	GC content (%)	*N*_50_ (bp)	No. of reads	Total no. of bases	Sequence coverage (×)	Assembly accession no.	SRA accession no.
E. coli	2215	599	76	5,194,917	50.46	229,671	7,849,332	792,782,532	152.61	JACAXQ000000000	SRR12056276
E. coli	7856	131	87	5,278,546	50.86	166,652	7,796,846	787,481,446	149.19	JACAXP000000000	SRR12056275
E. coli	9782	372	64	5,290,892	50.58	189,391	8,184,758	826,660,558	156.24	JACAXO000000000	SRR12056274
K. pneumoniae	10376	664	76	5,527,810	57.17	193,177	7,878,948	795,773,748	143.96	JACAXN000000000	SRR12056273
K. pneumoniae	9302	429	92	5,929,747	56.56	203,934	7,744,516	782,196,116	131.91	JACAXM000000000	SRR12056272
K. pneumoniae	10332	664	79	5,525,694	57.17	185,570	7,393,420	746,735,420	135.14	JACAXL000000000	SRR12056271
K. pneumoniae	10830	34	120	5,776,072	56.98	165,271	7,635,060	771,141,060	133.51	JACAXK000000000	SRR12056270
K. pneumoniae	11366-3	628	59	5,665,193	57.18	218,486	5,781,576	583,939,176	103.07	JACAXJ000000000	SRR12056269
K. pneumoniae	1830-2	327	139	5,677,045	57.11	111,734	7,169,824	724,152,224	127.56	JACAXI000000000	SRR12056268
K. pneumoniae	8657-2	1123	78	5,648,280	57	181,873	12,071,670	1,219,238,670	215.86	JACAXH000000000	SRR12056267

a The sequence data of all isolates have been deposited in GenBank under BioProject accession number PRJNA640587.

b MLST, multilocus sequence type.

These E. coli and K. pneumoniae assemblies provide additional insight into the genomic diversity of Gram-negative bacteria that harbor the FOX-5 β-lactamase-encoding gene and were retrieved from an ICU surveillance cohort.

### Data availability.

All data have been made publicly available and were deposited under the accession numbers listed in Table 1.
